# BUFET: boosting the unbiased miRNA functional enrichment analysis using bitsets

**DOI:** 10.1186/s12859-017-1812-8

**Published:** 2017-09-06

**Authors:** Konstantinos Zagganas, Thanasis Vergoulis, Maria D. Paraskevopoulou, Ioannis S. Vlachos, Spiros Skiadopoulos, Theodore Dalamagas

**Affiliations:** 1 University of Peloponnese, Department of Informatics and Telecommunications, Tripoli, 22100 Greece; 2 “Athena” Research and Innovation Center, Athens, 15125 Greece; 3 DIANA-Lab, Department of Electrical & Computer Engineering, University of Thessaly, Volos, 38221 Greece; 4 Hellenic Pasteur Institute, 127 Vasilissis Sofias Avenue, Athens, 11521 Greece

**Keywords:** miRNAs, Functional enrichment analysis, BUFET

## Abstract

**Background:**

A group of miRNAs can regulate a biological process by targeting genes involved in the process. The unbiased miRNA functional enrichment analysis is the most precise *in silico* approach to predict the biological processes that may be regulated by a given miRNA group. However, it is computationally intensive and significantly more expensive than its alternatives.

**Results:**

We introduce BUFET, a new approach to significantly reduce the time required for the execution of the unbiased miRNA functional enrichment analysis. It derives its strength from the utilization of efficient bitset-based methods and parallel computation techniques.

**Conclusions:**

BUFET outperforms the state-of-the-art implementation, in regard to computational efficiency, in all scenarios (both single- and multi-core), being, in some cases, more than one order of magnitude faster.

## Background

microRNAs (miRNAs) are short (∼23nt) non-coding RNA molecules that are considered to be central gene expression regulators. They act through mRNA degradation and/or translational suppression of protein coding transcripts. By binding to specific recognition elements with perfect or imperfect base complementarity, miRNAs interact with genes and inhibit their expression. Consequently, they can play a key role in the regulation of numerous biological processes and, thus, miRNA-induced up- or down-regulation can be indicative of a diseased state [1–3]. On the other hand, each miRNA can target hundreds of different genes [4] and its perturbed expression can, in turn, affect numerous biological functions. This makes the analysis of the effects of miRNAs on biological processes crucial to the understanding of this post-transcriptional regulation mechanism.

*miRNA functional enrichment analysis* is the *in silico* process which enables researchers to discover potential biological functions affected by a group of differentially expressed miRNAs. The first step of this process is the identification of all genes targeted by at least one of the miRNAs in the group. In most cases, these gene sets are produced by target prediction algorithms like DIANA-microT [5, 6], miRanda [7] or TargetScan [8]. Then, gene annotation data (e.g., pathways, functions, etc) for all known genes are collected. These data are usually retrieved by the Gene Ontology (GO) Consortium [9] (or other sources like KEGG [10, 11] and PANTHER [12]) and capture the involvement of genes in several biological processes. Finally, a statistical analysis is applied on the data collected during the previous two steps, to reveal the annotation categories that are overrepresented in the genes targeted by the miRNA group. Usually, the algorithm selected for this step is Fisher’s exact test [13, 14], which calculates *p*-values based on the hypergeometric distribution.

However, it has recently been shown [15] that the use of the aforementioned statistics approach can produce significant *p*-values even for biological processes, controlled by groups of randomly selected miRNAs. This indicates that an underlying bias exists between miRNAs, their predicted gene-targets and the structure of the annotation, also reflected in the performed enrichment analyses. Thus, in order to overcome this problem, the authors of [15] proposed a Monte Carlo test which produces an *empirical p-value*. Moreover, as pointed out by the authors of [16], this approach moves the analysis from the gene to the miRNA level by defining the *biological process overlap* as the proportion of those genes that are both targeted by the miRNA group of interest and also involved in the biological process under examination. In brief, their approach is the following: first, a large number of randomly assembled miRNA groups having the same number of miRNAs as the group of interest are selected. Then, the empirical *p*-value is defined as the proportion of those random groups that exhibit a greater biological process overlap than the miRNA group under examination. More details on the benefits of this approach to miRNA functional enrichment analysis are available in the original paper [15] by Bleazard et al.

The number of random miRNA groups selected to perform the analysis is a parameter that controls the *accuracy* of the *p*-value to be produced. In particular, the higher the number of random miRNA groups selected, the more accurate the produced *p*-value will be. Usually, 1 million random groups are used to achieve sufficient accuracy [15]. Unfortunately, using such a large number of groups results in unreasonably large execution times. For example, an execution of the state-of-the-art implementation [15] for a group of 100 miRNAs as input, using 1 million random groups, on a single core of an Intel i7-3820 processor requires up to 17 h of processing time.

In order to alleviate this issue, we introduce BUFET (Bitset-based Unbiased miRNA Functional Enrichment Tool). This approach exploits efficient data structures to significantly reduce the execution time of the unbiased enrichment analysis. BUFET also takes advantage of parallel computing techniques to achieve additional performance improvements in multi-core systems. The contribution of this work can be summarized in the following:

- We studied the computational requirements and examined the performance bottlenecks of the unbiased miRNA functional enrichment analysis.
- We investigated the performance of different data structures, namely hash tables and bitsets, in regards to their effectiveness in unblocking the identified bottlenecks.
- We developed BUFET, a tool that utilises the results of the aforementioned investigation to boost the speed of the unbiased miRNA functional enrichment analysis. To achieve an even greater speed boost in the case of multi-core environments, we exploited multithreading to implement parallel execution of the analysis.
- We performed an extensive evaluation of BUFET to demonstrate its efficiency. BUFET outperforms the state-of-the-art approach in all scenarios (in many cases by an order of magnitude).
- We provide BUFET as an open source implementation, which is freely available on GitHub (see the “Availability and requirements” section). BUFET is a powerful tool that provides flexible input file formats enabling many execution modes (e.g., execution using custom miRNA-gene interactions and gene annotations).

## Implementation

### The challenge

As mentioned previously, the unbiased miRNA functional enrichment analysis involves the examination of a large number of biological processes (or, equivalently, annotation categories) to identify those, which are more likely to be affected by the gene-targets of a miRNA group. During this type of analysis, both biological processes and miRNAs are represented as gene sets: each biological process is represented by the genes involved in it, while each miRNA by its gene-targets.

It becomes evident that computing the biological process overlap of a miRNA group (see the “Background” section) involves the calculation of the *intersection* between the set of genes targeted by the miRNA group and the set of genes involved in the biological process. Moreover, the set of genes targeted by each miRNA group needs to be calculated “on the fly” by performing *union* operations on the gene sets of each miRNA in the group. Therefore, the unbiased miRNA functional enrichment analysis relies on performing a very large number of set unions and intersections. For instance, for a given query miRNA group of size 10, about 10 million unions and more than 8 billion intersections are required to produce a *p*-value.

The state-of-the-art implementation of the unbiased miRNA functional enrichment analysis [15] uses hash tables (more specifically, Python sets^1^) to represent gene sets. The advantage of this data structure is that performing union and intersection operations for small sets is usually very fast. Both operations are performed by executing a variant of the hash-join algorithm [17]. On the other hand, hash-join becomes very inefficient when operating on large sets.

Unfortunately, in the case of the unbiased miRNA functional enrichment analysis, all union operations are performed on large gene sets. This is attributed to the fact that each of these gene sets corresponds to the predicted targets of a particular miRNA. Since miRNA target prediction algorithms usually produce hundreds or even thousands of results (interactions) for a single miRNA, it becomes evident that most of the performed union operations can be quite slow if hash-join is used.

To overcome this problem, the *bitset* (or *bit-vector*) [17], an alternative data structure, which is more suitable for the representation of large sets, can be used. When sets of genes are implemented as bitsets, unions and intersections between them can be calculated by performing bitwise operations on bit blocks. In particular, bitwise-or can be used to get the union of two sets, while bitwise-and to get their intersection. Such operations are efficient for large sets, since their execution time is not affected by the size of the set^2^. Additionally, the representation of gene sets as bitsets is more efficient, memory-wise, in the case of relatively large sets of genes (like those produced by miRNA target prediction algorithms).

The calculation of the targets of each miRNA group would benefit greatly by the use of bitwise-or, since, as previously mentioned, it involves a large number of union operations on large gene sets represented by dense bitsets. In this case, bitsets also have a reduced memory footprint compared to hash-tables. On the other hand, gene sets related to biological processes, as provided by Gene Ontology annotations [9], usually consist of a small number of genes. Therefore, hash-join on these sets can be rather efficient^3^.

The previous discussion suggests that a *hybrid solution*, using bitwise operations for unions and hash-join for intersections, seems more suitable than both of the aforementioned approaches. Unfortunately, this hybrid approach has a major drawback. The gene sets generated by bitwise-or for all miRNA groups must be provided as input to the hash-join algorithm for the calculation of the biological process overlaps. However, the bitwise-or algorithm produces gene sets represented as bitsets, while hash-join requires its input in the form of hash tables. Therefore, a data structure conversion must be performed, introducing an important execution overhead that counterbalances any gains in efficiency.

### The BUFET approach

**Fig. 1:**
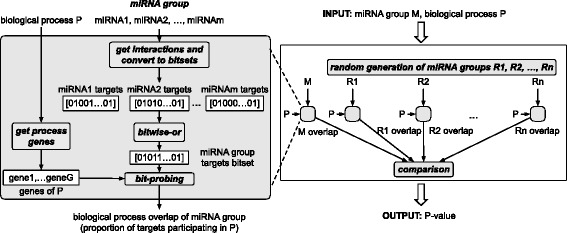
Flowchart summarizing the BUFET approach

Our approach, called BUFET, is demonstrated in Fig. 1. It combines the best characteristics of bitset- and hash-table-based methods without suffering from the aforementioned shortcomings of a hybrid approach. It takes advantage of the efficiency of bitwise-or in calculating the union of large sets to produce the gene sets targeted by particular miRNA groups. These gene sets are represented as bitsets, called *miRNA group bitsets*.

Meanwhile, the biological process overlap of each miRNA group is calculated as follows: for each gene annotated as part of the biological process, the respective bit in the miRNA group bitset is examined. If the bit is set, then the value of a counter is increased by one (its value is initially zero). Otherwise, the value of the counter remains intact. After all genes related to the biological process have been considered, the value of the counter provides the size of the intersection and, subsequently, the biological process overlap. Since the genes are used to probe the miRNA group bitset, we refer to this method as bit-probing.

Further optimizations were introduced in order to achieve additional performance improvements. First, biological processes that have no common genes with the miRNA group under examination can be excluded from the analysis (since no interference by the miRNAs in the group with the process is recorded). Additionally, BUFET supports full utilization of multi-core computing systems by supporting parallelization at the biological process level.

It should be noted that parallel execution is also supported by the state-of-the-art approach presented in [15]. However, in contrast to the use of multiprocessing adopted by this approach to implement parallelization, BUFET uses multithreading. The advantage of multithreading over multiprocessing is that all processes running in parallel have access to the same part of the main memory. This eliminates the need to copy data across processes, thus reducing the execution time and memory footprint.

On the other hand, an issue with this approach is that the bitsets containing the targets of the random miRNA groups have to be calculated and stored in main memory. This step is necessary, so that every thread is able to access the data in order to calculate a *p*-value. Consequently, this increases the memory footprint, although, the amount of memory required does not pose a big challenge for contemporary computers. More specifically, none of the many real-world analysis scenarios examined during our experiments resulted in the allocation of more than 3.5 GB of RAM to our script.

### Functionality and source code

BUFET is provided as a free, open source software licensed under GPL v3 (a download link is provided in the “Availability and requirements” section). Its core is implemented in C++ for greater efficiency, while a Python wrapper script facilitates its execution and its incorporation in existing bioinformatics workflows.

The input of the BUFET software consists mainly of two CSV files: one containing miRNA-to-gene interactions and another containing associations of biological functions with particular genes. The proper format of these files is described in the software download page. It should be noted that BUFET provides flexibility, enabling the users to upload miRNA-to-gene interactions based on the prediction algorithm of their choice (e.g., TargetScan [8], DIANA-microT [5, 6], miRanda [7], etc.) and to use biological function annotations collected by their preferred source (e.g., GO [9], KEGG [10, 11], or PANTHER [12]).

Finally, BUFET also performs *Benjamini-Hochberg FDR correction* [18]. More specifically, following the method in [19], we assume that 5% (and 1%) of the produced *p*-values (under the 0.05 threshold) are false positives, while the rest are significant results. *P*-values significant at FDR 0.05 are marked with “*” while *p*-values significant at 0.01 are marked with “**” in the output file.

## Results

In this section, the efficiency of BUFET is evaluated against that of the state-of-the-art implementation (EmpiricalGO ^4^), in both single- and multi-core environments. First, we examine the effect of the miRNA group size on the execution times of both implementations. Next, we investigate their parallel behavior for a varying number of CPU cores. miRNA-to-gene interactions were collected from DIANA-microT-CDS (score threshold=0.8) and miRanda (score threshold=155 and free energy= −20), while GO annotation data were obtained from Ensembl. Statistics related to miRNA-to-gene-interactions data used are presented in Table 1. All experiments were executed on a machine powered by an Intel Core i7-3820 processor with 8 cores (4 physical) and 64 GB of main memory.

**Table 1:** Statistics related to the miRNA-to-gene interactions used

	Number of genes/miRNA					Total miRNAs
	Minimum	Maximum	Average	Median	Std. Deviation	
microT	1	4547	404	206	459	2580
miRanda	11	6977	1309	1096	932	2588

### Varying the miRNA group size

**Fig. 2:**
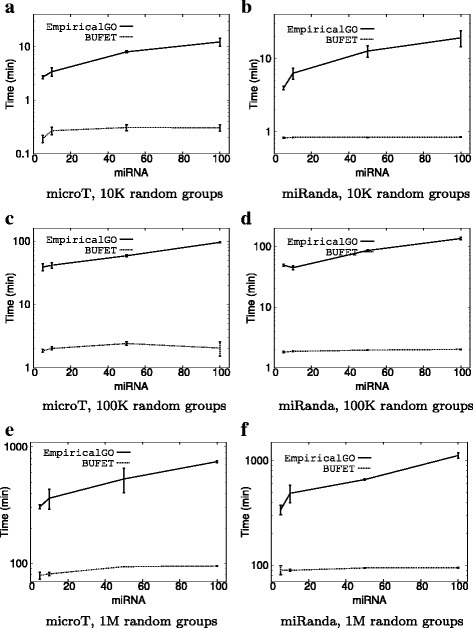
Average execution times (log scale) on a single core with a varying number of miRNAs. (**a**) microT, 10K random groups. (**b**) miRanda, 10K random groups. (**c**) microT, 100K random groups. (**d**) miRanda, 100K random groups. (**e**) microT, 1M random groups. (**f**) miRanda, 1M random groups

Figure 2 presents (a) the average execution time of BUFET and EmpiricalGO and (b) its standard deviation (using error bars) for each measurement point and for varying miRNA group sizes (5, 10, 50 and 100 miRNAs) in a single-core environment. For each miRNA group size, 10 different groups were used as input to both implementations. Thus, every reported execution time is the average of 10 executions. The left column corresponds to the experiment performed using DIANA-microT-CDS interactions, while the right to the one using miRanda interactions. We performed each experiment by selecting the following, commonly-used settings: 10 thousand (10K), 100 thousand (100K), and 1 million (1M) random miRNA groups. Since the difference in the execution times between EmpiricalGO and BUFET are very large, all diagrams are presented in log scale for the y axis to enhance legibility.

It is clear that the execution time increases as the number of miRNAs in the group under examination increases for both approaches (due to the larger number of union operations that have to be performed). However, it is evident that the rate of the increase in the execution time is larger for EmpiricalGO than BUFET. This can be attributed to the fact that BUFET exploits the efficiency of bitwise-or in calculating unions on large gene sets. It also becomes evident that BUFET scales better than EmpiricalGO and in some cases, it is faster by at least an order of magnitude. Therefore, BUFET is a very efficient approach when high accuracy is needed for functional analysis of large miRNA groups.

**Fig. 3:**
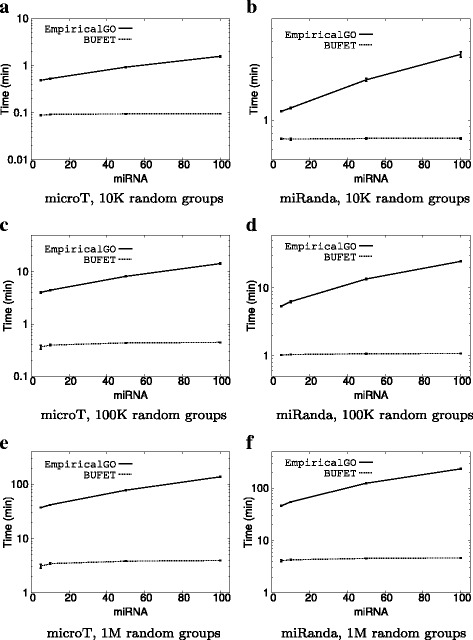
Average execution times (log scale) on 7 cores with a varying number of miRNAs. **a** microT, 10K random groups. **b** miRanda, 10K random groups. **c** microT, 100K random groups. **d** miRanda, 100K random groups. **e** microT, 1M random groups. **f** miRanda, 1M random groups

Figure 3 shows the same experiments in a multi-core environment (7 cores were used). Note that the main trends observed in the single-core experiment continue to occur: increasing the miRNA group size leads to increased execution times for both methods, while BUFET is significantly more efficient than EmpiricalGO in all cases. Note that, for the case of 5 miRNAs in low accuracy mode, the execution times tend to converge to the time needed for serial operations (i.e. file reading, output writing, and FDR correction). Finally, it is worth mentioning that, in the case of 100 miRNAs using 7 cores, in high accuracy mode, BUFET can produce results in under 5 min, while EmpiricalGO needs more than 7 h for the samel task.

### Varying the number of cores

**Fig. 4:**
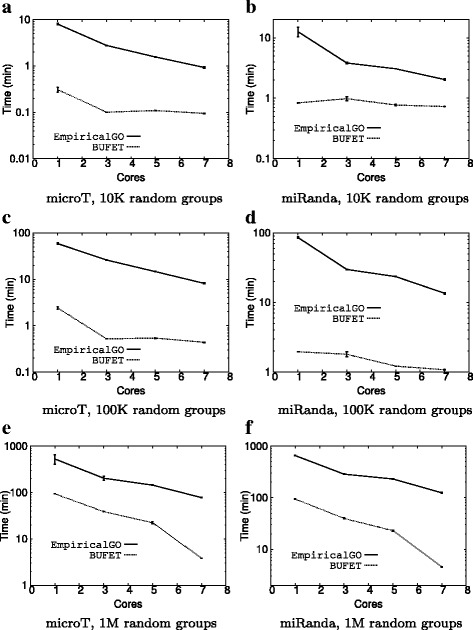
Average execution times (log scale) varying the number of cores. **a** microT, 10K random groups. **b** miRanda, 10K random groups. **c** microT, 100K random groups. **d** miRanda, 100K random groups. **e** microT, 1M random groups. **f** miRanda, 1M random groups

Figure 4 shows the average time required by BUFET and EmpiricalGO to calculate the empirical *p*-values for 10 input groups of size 50 by using a varying number of CPU cores. It is clear that both approaches become faster as the number of cores increases. However, in every case BUFET requires significantly less time to execute.

## Conclusion

In this paper we dealt with the performance of the unbiased miRNA functional enrichment analysis. We showed that the state-of-the-art approach to perform this type of analysis (EmpiricalGO) is not practical in terms of computational efficiency, especially for large miRNA groups when high accuracy is required. To deal with this problem we introduced BUFET, an alternative bitset-based approach. Our experiments make evident that BUFET outperforms the state-of-the-art implementation in all scenarios (in many cases by orders of magnitude). Additionally, the better scalability of BUFET makes it a very appealing solution for the analysis of large miRNA groups when 1 million random groups are used for the analysis. Note that, BUFET is provided as an open source implementation which is freely available on GitHub (the download URL is provided in the “Availability and requirements” section).

## Availability and requirements

**Project name**: BUFET

**Project home page**: https://github.com/diwis/BUFET/

**Operating system(s)**: Linux, MacOSX.

**Programming language**: C++, Python.

**Other requirements**: Python interpreter 2.7 or higher, g++ 4.8 or higher.

**License**: GNU GPL v.3.

**Any restrictions to use by non-academics**: None

## Endnotes

^1^ https://docs.python.org/3/tutorial/datastructures.html

^2^ In particular, the execution time of each bitwise operation depends on the number of bits it contains, i.e., on the cardinality of the set’s domain.

^3^ Regarding the calculation of the biological process overlap, an additional optimization is possible for the hash-join algorithm. In particular, the production of the output intersection set can be avoided, since only its size is required. However, a similar optimization is not feasible for bitwise-and.

^4^ http://sgjlab.org/empirical-go/

## Abbreviations

BUFET: Bitset-based unbiased functional enrichment tool
CPU: Central processing unit
CSV: Comma-separated values
FDR: False discovery rate
GB: Gigabytes
GO: Gene ontology
GPL: General public licence
KEGG: Kyoto encyclopedia of genes and genomes
miRNAs: microRNAs
PANTHER: Protein ANalysis THrough evolutionary relationships
RAM: Random access memory
URL: Uniform resource locator
